# Improving institutional research ethics capacity assessments: lessons from sub-Saharan Africa

**DOI:** 10.1080/11287462.2018.1528660

**Published:** 2018-10-04

**Authors:** Molly Deutsch-Feldman, Joseph Ali, Nancy Kass, Nthabiseng Phaladze, Charles Michelo, Nelson Sewankambo, Adnan A. Hyder

**Affiliations:** a Department of Epidemiology, Johns Hopkins Bloomberg School of Public Health, Baltimore, USA; b Johns Hopkins Berman Institute for Bioethics, Baltimore, MD, USA; c University of Botswana, Gaborone, Botswana; d University of Zambia, Lusaka, Zambia; e Makerere University College of Health Sciences, Uganda; f Department of International Health, Johns Hopkins Bloomberg School of Public Health, Baltimore, USA

**Keywords:** Research ethics, LMIC, capacity assessment

## Abstract

The amount of biomedical research being conducted around the world has greatly expanded over the past 15 years, with particularly large growth occurring in low- and middle-income countries (LMICs). This increased focus on understanding and responding to disease burdens around the world has brought forth a desire to help LMIC institutions enhance their own capacity to conduct scientifically and ethically sound research. In support of these goals the Johns Hopkins-Fogarty African Bioethics Training Program (FABTP) has, for the past six years, partnered with three research institutions in Africa (University of Botswana, Makerere University in Uganda, and the University of Zambia) to support research ethics capacity. Each partnership began with a baseline evaluation of institutional research ethics environments in order to properly tailor capacity strengthening activities and help direct limited institutional resources. Through the course of these partnerships we have learned several lessons regarding the evaluation process and the framework used to complete the assessments (the Octagon Model). We believe that these lessons are generalizable and will be useful for groups conducting such assessments in the future.

## Introduction

Over the past few decades, biomedical research has become increasingly globalized, including a remarkable increase in biomedical research conducted in low- and middle-income countries (LMICs) (Glickman et al., 2009; Moses et al., 2015). This growth has included an expansion of research conducted across African institutions (Ndebele et al., 2014; Schemm, 2013). To support this work, groups such as the UK Wellcome Trust have provided funding to support research projects and biomedical research training for scientists across the continent (The Wellcome Trust, 2013, 2014). Pharmaceutical companies such as Merck and Novartis have also contributed to the growing global research environment and provided funding to help establish research centers in Africa (The Merck Group, 2014; University of Cape Town Department of Marketing and Communication, 2014).

Several organizations have also supported programs to foster strengthening of research *ethics* capacity, particularly within LMICs. In 2011, the European & Developing Countries Clinical Trial Partnership (EDCTP) provided 49 grants to countries in sub-Saharan Africa to support research ethics infrastructure, including Research Ethics Committees (RECs) (European & Developing Countries Clinical Trials Partnership, 2011). Between 2000 and 2012, The Fogarty International Center of the U.S. National Institutes of Health (NIH-FIC) invested resources in programs to strengthen research ethics capacity across the world; nearly a third of which went specifically to programs in sub-Saharan Africa (Ndebele et al., 2014; Saenz et al., 2014). This funding has supported, primarily, international research ethics curriculum development (Saenz et al., 2014). As of 2016, NIH-FIC is funding 22 research ethics training programs across the world, all of which are conducted within LMICs (The Fogarty International Center, 2016). Groups such as the Medical Education Partnership Initiative, the African Malaria Network Trust, and the Training & Resources in Research Ethics Evaluation group also work to provide research ethics training, with a specific focus on supporting African researchers (AvacNet, 2016; The Fogarty International Center, 2017; Training and Resources in Research Ethics Evaluation, 2016).

As international research and ethics training programs increase and evolve, efforts to evaluate and monitor the impact of such programs have emerged, though few empirical tools exist for this purpose (Ali, Kass, Sewankambo, White, & Hyder, 2012; Millum, Grady, Keusch, & Sina, 2016). Most past methods of research ethics capacity assessment have focused mainly on determining impact upon individuals (Ajuwon & Kass, 2008; Kass, Ali, Hallez, & Hyder, 2016) or on specific programs such as Internal Review Boards (Sidle et al., 2006). However, there is also a need to examine LMIC research ethics capacity at the institutional level. In an effort to support this level of assessment, the Johns Hopkins-Fogarty African Bioethics Training Program (FABTP) has developed a systems approach to evaluating institutional research ethics capacity. This involves evaluating the research institution as a whole, particularly focusing on areas such as faculty training, ethics coursework for students, ethics policies, research ethics financing, and the functioning of research ethics infrastructure (e.g. IRBs) and programs. Others have conducted institutional research ethics needs assessments; though not guided by a formal systems framework (Sidle et al., 2006). A systems approach evaluates not only individual capacities, but also university-level commitments and the research environment in which research ethics programs operate.

Since 2010, we have conducted three institutional research ethics capacity assessments at universities in eastern and southern Africa. These evaluations were conducted collaboratively with the University of Botswana, Makerere University College of Health Science in Uganda, and the University of Zambia (Hyder et al., 2013, 2015, 2017). To complete these assessments, we developed several qualitative and quantitative data collection tools and used a modified version of the Octagon Model (“the Octagon”), first developed by the Swedish International Development Agency (SIDA) to analyze organizations (Swedish International Development Cooperation Agency, 2002). We chose the Octagon because of its: flexibility of application (in terms of time frames), ease of use (in terms of operations), and its multi-dimensional nature. We also note again that we found a lack of specific models for the aims of our work - thus allowing us to contribute this approach to the field. We adapted the model for our use by using the original eight domains of the model, but tailoring the questions to each university’s research ethics program (Table 1).

**Table 1.** Overview of domains and components of the Octagon model as adapted for institutional research ethics capacity evaluation.

Domain	Example components
Basic values and identity	Does the institution have clearly stated research ethics goals and objectives?
Structure and organization of activities	Are the roles and responsibilities clear for each member of the institution? Are members aware of these roles?
Implementation of activities	Are the operational plans clearly defined? Is there a system in place to determine programmatic strengths and weaknesses?
Relevance	Do the programs in place actually help the institution accomplish its goals?
Right skills in relation to activities	Are institutional personnel involved in research ethics capacities qualified for such roles?
Systems for financing and administration	Is there adequate funding for the institution to accomplish its research ethics goals?
Target groups	Are target groups clearly identified and actively engaged with research ethics as relevant to their institutional roles?
Working environment	What role does the institution play in the larger research ethics context (locally, nationally or internationally) of which it is a part?

Note: Adapted from: Swedish International Development Cooperation Agency. *The Octagon: A Tool for the Assessment of Strengths and Weaknesses in NGOs*; 2002.

Several generalizable lessons regarding the assessment framework and its application have emerged from these evaluations and in this paper we describe our experience with research ethics system evaluation so others may learn from and improve upon the analytic approach. We aim to aid those at research institutions hoping to conduct such assessments; this tool can be used by external colleagues, internal teams, or a combination. It is also flexible so that it can be used as a baseline or interim or final evaluation assessment. We also present a comparison of the Octagon with a framework from the ESSENCE on Health Research group, a model developed by the Special Programme for Research and Training in Tropical Diseases (ESSENCE on Health Research, 2014). The ESSENCE framework provides seven principles to guide research capacity building in LMICs, and while commonly used may not necessarily provide the proper structure for all program development. The comparison demonstrates the Octagon’s applicability as a complementary model for strengthening research ethics capacity.

We begin with a brief overview of the goals of institutional research ethics capacity development, provide a summary of the three previously conducted case studies, discuss our evaluation process, describe lessons learned regarding the evaluation process, present the comparison with ESSENCE, and discuss areas for future growth of the model.

## Institutional research ethics capacity assessment

**Figure 1.**
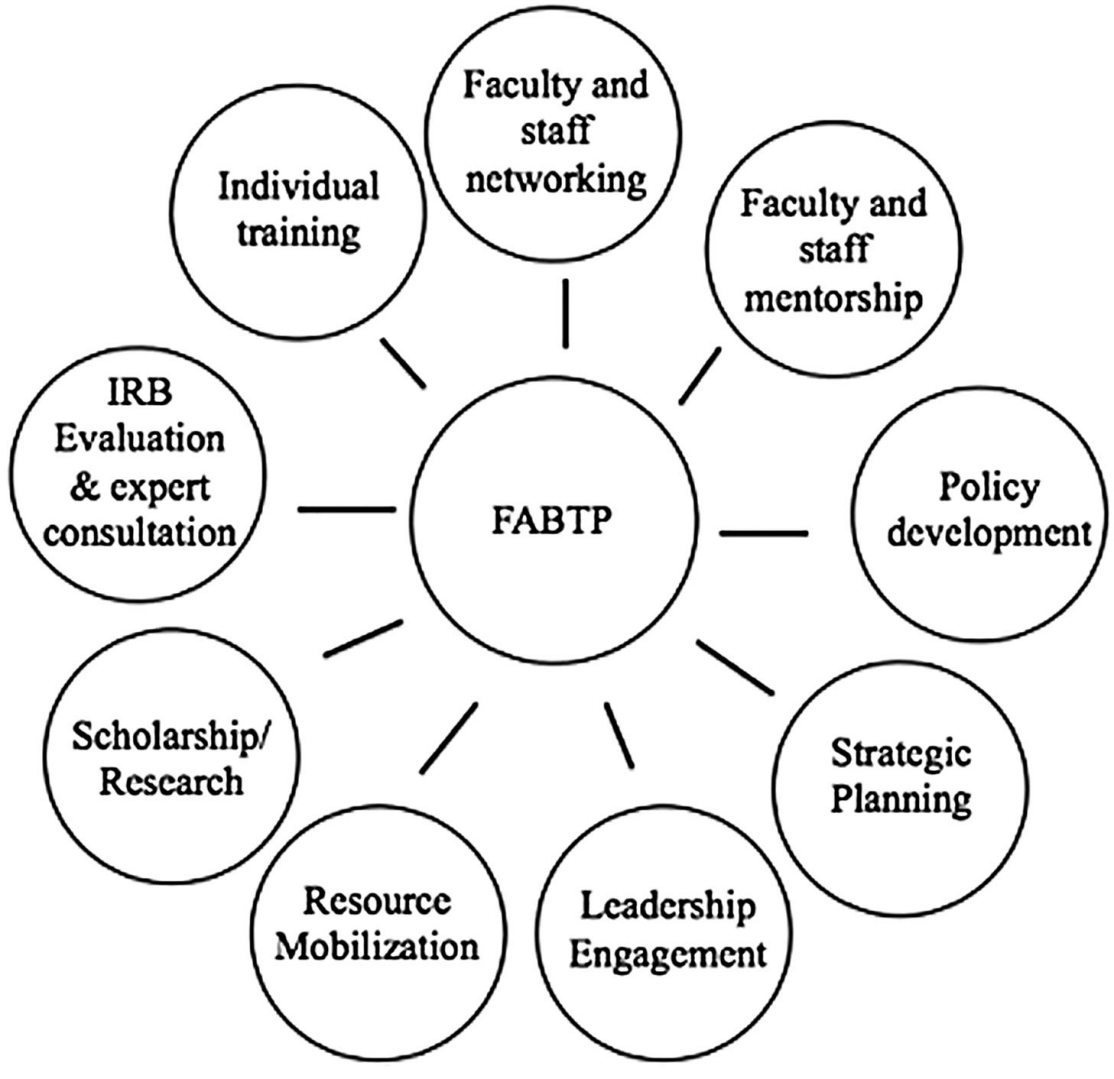
Components of the FABTP research ethics system strengthening approach.

With funding from the NIH-FIC, the Johns Hopkins Fogarty African Bioethics Training Program (FABTP) engages in research ethics partnerships with institutions across Africa in order to support strengthening of institutional research ethics capacity. The development of FABTP has been described previously (see Hyder et al., 2013, 2015). FABTP aims to provide a multi-faceted, holistic approach to research ethics program development, in close collaboration with, and guided by, the objectives of partner institutions. This broad approach to research ethics capacity development can often serve LMIC well, where an underlying research ethics infrastructure may not be present or resources for ethics development are limited. Core partnership activities include strategic planning, leadership engagement, short- and long-term training, policy development, and collaborative research (Figure 1).

A critical element of the FABTP approach is system evaluation, conducted at the beginning of the institutional partnership. This allows FABTP to determine potential areas of strength and for growth within the institutions’ existing research ethics infrastructure. The assessment is meant to serve as a foundation to guide and benchmark future activity and development.

A modified version of the Octagon Model provided a framework to guide institutional assessment. The Swedish International Development Agency (SIDA) first developed this approach as a method for evaluating non-governmental organizations (Swedish International Development Cooperation Agency, 2002). The model assesses institutions using eight domains, which we adapted for our purposes in order to apply the Octagon to university research ethics systems. The domains used in each of the three institutional assessments were: basic values and identity, structure and organization, implementation, relevance, proper skills, system for financing and administration, target groups, and working environment. We have previously detailed each of these domains, and a brief summary is presented in Table 1 (Hyder et al., 2013, 2015, 2017). Each domain is ranked on a scale of 1 through 7 to generate an overall Octagon score. As stated, we used the original Octagon framework as designed by SIDA, but adapted the questions to focus on each university’s research ethics capacity.

Data were collected using mixed methods, and a standardized approach was adapted for different sites. In Botswana and Uganda, we conducted semi-structured in-depth interviews (IDIs) with key informants such as deans and deputy vice chancellors. We also conducted focus group discussions (FGDs) with students, research faculty and staff, and IRB members (each of these groups were interviewed separately) (Hyder et al., 2013, 2015). Due to time constraints, we were unable to conduct IDIs and FDGs in our Zambia evaluation (Hyder et al., 2017). However, all three institutional assessments included administration of structured questionnaires to capture detailed information from university and IRB leadership responsible for local research systems and ethics oversight. The questionnaires contained 168 questions covering a range of topics including available research ethics coursework, training for IRB members, and finances. All assessments also included informal discussions with staff members and local research ethics faculty, as well as a review of pertinent institutional documents (e.g. research/ethics policies, strategic plans, standard operating procedures). Data from all sources is used to generate final Octagon scores.

Once data are cleaned, verified and reviewed, a standardized process should be followed for generating numerical scores across the eight domains of the Octagon. SIDA provides a guideline for creating scores; however, final scores are generated based on team assessments of available information. For each case study, two individuals generated scores for each domain. Like other deliberative tools, the scoring is essentially subjective and dependent on the team that conducts the assessment. Though lower scores indicate areas of most need, small differences in scores make it difficult to decide in which areas institutions should focus their efforts. Octagon scores are not meant to be compared across institutions, since the evaluation is largely influenced by the political and cultural context of the institution. Understanding these limitations is necessary for properly interpreting and utilizing the results of the Octagon.

**Figure 2:**
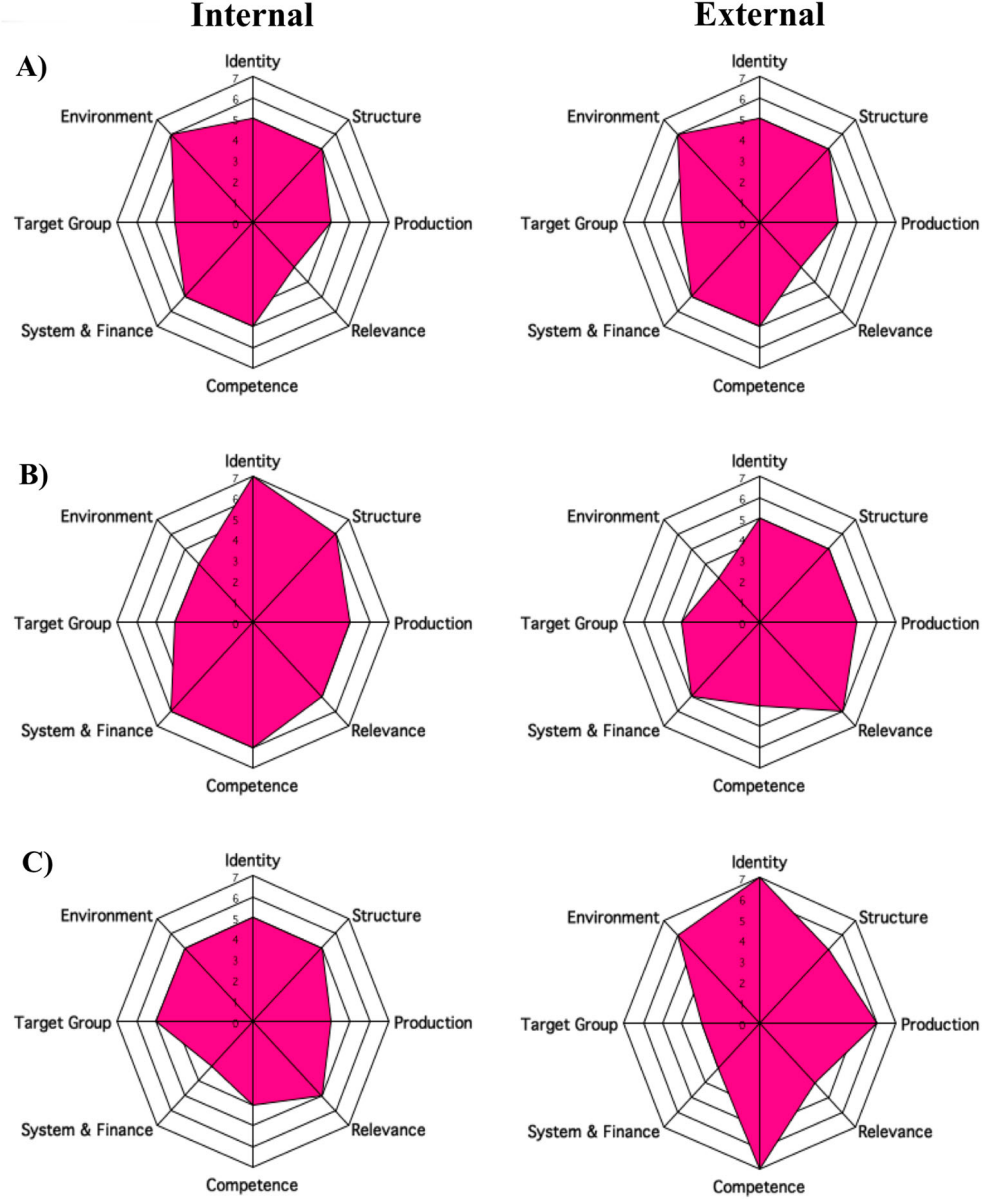
. Octagon scores from three case studies. A) Zambia B) Botswana C) Uganda. Institutional self-evaluation (internal) scores on left, FABTP (external) scores on right.

To create a more balanced score system and reduce the subjectivity of the scoring, we generated both internal (from those within the university) and external (from the staff at Johns Hopkins University) scores for all three institutional assessments (Figure 2). Four individuals (two from the institution for the internal score and two from FABTP for the external score) independently scored each domain of the Octagon. Final internal and external scores were calculated by averaging the two individual scores. However, to further reduce potential bias, we recommend at least three individuals from the institution generate the internal score, and three separate individuals generate the external score.

In addition to the Octagon scores, we suggest that evaluations aim to include other forms of feedback. These include conversations with university faculty and reports of key strengths and challenges for each domain of the Octagon. These provide a more in-depth analysis that aid the institutional staff as they work to further build research ethics capacity (Hyder et al., 2013, 2015, 2017).

## Overview of three case studies

As stated, FABTP has completed three cases studies to date. Each involved a partnership between the institution and Johns Hopkins University to conduct a baseline capacity assessment as well as a needs assessment. Each case study has been published, detailing the implementation of the assessment as well as specific areas of need for each institution (Hyder et al., 2013, 2015, 2017). The first case study was conducted at the University of Botswana in Gaborone, Botswana beginning in 2010 (Hyder et al., 2013). The second was conducted in 2011 at Makerere University College of Health Sciences in Kampala, Uganda (Hyder et al., 2015). The last case study was conducted in 2013 as a “rapid assessment” with the University of Zambia in Lusaka, Zambia (Hyder et al., 2017). The Octagon figures from all three case studies are presented in Figure 2.

## Lessons learned

Through the process of adapting an approach for institutional assessment to the research ethics context across three institutions, several lessons emerged for further discussion and research. Some relate to the integration of a systematic data collection approach within an institutional capacity development partnership; others relate more specifically to the data collection tools and model. We emphasize that the components of an institutional evaluation (overall assessment approach and analysis) must have key characteristics to be most effective, relevant, and supportive of meaningful institutional growth. The strengths and limitations of the Octagon Model are explored in view of these critical factors (Table 2). We present these lessons to demonstrate how the Octagon may be used by others for future assessments.

**Table 2.** Characteristics of the Octagon Model applied in each of the case studies.

Characteristics of model	Case study		
	Botswana	Uganda	Zambia
Locally relevant	Yes	Yes	Yes
Timely	Three years between data collection and completion of evaluation	Three years between data collection and completion of evaluation	Three years between data collection and completion of evaluation
Multi-dimensional	Yes	Yes	Yes
Participatory	Yes	Yes	Didn’t include students and other key stakeholders
Empirically based	First study from FABTP – relied on previous verification and published studies	Yes - One previous case study from FABTP	Yes - Two previous studies from FABTP
Multi-method	Yes	Yes	Relied almost entirely on structured questionnaire and site visits
Iterative	Yes	Yes	Yes

Note: Adapted from Hyder et al. (2013, 2015, 2017).

### Overall assessment approach

The evaluation process serves both as a baseline capacity assessment and a needs assessment. These are critical as they determine the starting point and course of action for future capacity development. There are several characteristics that help ensure an effective assessment including: contextual relevance, participation, multi-dimensionality, and the ability to monitor change over time.

In order to conduct an assessment that addresses the research ethics capacity and needs of an institution such as a university, that serves a public “good,” the evaluation must consider the larger research and policy context in which the institution operates. The assessment should also aim to be locally relevant by including the views of both internal and external stakeholders. This will help decrease potential bias and ensure that future capacity strengthening efforts align with the needs of those within the broader community of stakeholders. In our three assessments, we spoke formally with many different individuals within each university: students, research staff, administrative leadership and IRB members; and informally with some external stakeholders, such as researchers who collaborate with the institutions under assessment. Future assessments may benefit from including the perspectives of additional external stakeholders such as donors and governmental officials.

Ensuring participation by members of the university community is another key aspect of a successful evaluation. The assessment is meant to reflect the opinions and priorities of those within the institutional research environment; it cannot be completed by objective observations alone. Thus, any evaluation should aim to include a variety of university personnel including administrative leadership, academic researchers, staff, and students. Additionally, these groups likely have different views regarding institutional research ethics capacity as their interests may differ according to roles and responsibilities within university and research contexts. The Uganda and Botswana case studies were able to include many of these groups (Hyder et al., 2013, 2015); however, the Zambia case study was limited to the formal input of a relatively small group of university faculty (Hyder et al., 2017). While informal discussions were able to enhance the data to a degree, other groups, most notably students, were left out; this limited our ability to conduct as comprehensive an assessment.

Evaluators should aim to use multiple methods in order to capture different forms of information. In our previous evaluations we used in-depth interviews, focus groups, reviewed key documents and administered questionnaires; informal conversations provided the opportunity for discussing clarifications, where necessary. Using individual interviews and focus groups in particular, we learned individual opinions and perceptions regarding research ethics programs, policies and entities; we were also able to discuss plans for future research ethics development. Structured questionnaires aimed at determining facts pertaining to the characteristics of the institution’s ethics policies, infrastructure, funding sources, and academic programs, amongst others. Together, these varied forms of data collection provided a well-rounded picture of existing research ethics capacity and goals for further development within the institutions.

To determine whether research ethics capacity has been improved, one must monitor the change over time; therefore, each component of the evaluation must be conducted more than once. In the three case studies, data were collected once at the beginning as a baseline to serve as a starting point for research ethics capacity development. It will be critical to conduct follow up assessments to determine the degree to which programmatic changes have affected institutional capacity.

### Analysis

An analytic tool used to conduct both baseline and needs assessments ought to have certain properties in order to effectively evaluate institutional research ethics capacity in a way which is meaningful and useful to the partnering institution. These include using previously validated analysis methods, multi-dimensionality, and timeliness; we explored these properties through our use of the Octagon Model.

Evaluations should be conducted using verified and tested analytic methods. The Octagon Model has been used and tested on numerous occasions since its creation in 1999 to assess a range of different organizations and programs including those focused on health promotion and social development (Bergfelt, Beier, & Ljungros, 2008; Ibragimova, 2010). These studies demonstrate the versatility of the framework. Our group has now used the model in three different case studies, providing further evidence of its utility in the context of research ethics.

The questionnaires we used were based on previously conducted research and modified over the course of their use in order to be most useful. FABTP staff developed their survey using previous work conducted by the authors as well as an assessment of national health research infrastructure from the World Health Organization (D’Souza & Sadana, 2006; Hyder et al., 2008). The questions were reviewed and updated after each use of the survey; questions that proved unhelpful, or for which answers were not easily obtainable, were dropped or reworded.

Ideally, system assessments will adopt a multi-dimensional approach in order to properly evaluate all aspects of an institution’s research ethics capacity. To this end, the Octagon Model proved useful as it lent itself to mixed methods data collection across eight different domains of research ethics capacity (Table 1). These domains provided a picture of not only of research ethics programs but also the university’s overall capacity to sustain such programs. They also enabled us to assess the degree to which research ethics is integrated within the research environment of the institutions.

A key element of data collection and analysis is timeliness. Since evaluations are conducted with the goal of being relevant to future collaborations and capacity strengthening activities, it was critical that each proceeded in a reasonable amount of time. A long period of data collection also makes completing the analysis more difficult. Our case studies in Uganda and Botswana took one year, however, while the initial data collection for the Zambia case study began in 2012, the analysis was not completed until 2015 (due to delays in obtaining data, personnel changes, and changes in university leadership) (Hyder et al., 2017). A three-year gap between data collection and analysis may make the needs assessment less useful; university staff or funding may have changed making the recommendations less relevant. Creating a timeline with clear deadlines before beginning data collection may aid this process.

Lastly, to ensure that institutions are working towards implementing changes, it is critical to monitor progress over time. One notable advantage of the Octagon is the flexibility for follow-up evaluations and comparison over time. In each of our three case-studies, follow-up was conducted through conversations with collaborators, and in one case it has resulted in specific focus on evaluation of the institutional review board (ethics committee) performance.

## Comparison with ESSENCE

We further assessed our approach by comparing it to a framework developed by the Special Programme for Research and Training in Tropical Diseases (ESSENCE on Health Research, 2014). The ESSENCE framework was developed in 2014 and provides seven principles to guide research capacity development in LMICs (ESSENCE on Health Research, 2014). The ESSENCE principles are: network, collaborate, communicate and share experiences; understand the local context and accurately evaluate existing research capacity; ensure local ownership and secure active support; build in monitoring, evaluation and learning from the start; establish robust research governance and support structures, and promote effective leadership; embed strong support, supervision and mentorship structures; and think long-term, be flexible and plan for continuity (ESSENCE on Health Research, 2014). As stated, the ESSENCE framework is commonly used and provides a comparator that allows for a more informed assessment of the Octagon.

The similarities and differences between The Octagon and ESSENCE highlight how the two frameworks can be used in conjunction. There are areas of overlap between the two frameworks (Table 3). However, ESSENCE provides a guide for development, whereas the Octagon is a tool explicitly designed for evaluations. This difference is one reason why The Octagon is preferable for conducting institutional assessment. The Octagon approach also stresses the importance of engaging local stakeholders and understanding the context of the program. There is also an emphasis on establishing strong leadership. However, the ESSENCE framework supports long-term follow up and program monitoring, components that are not currently part of our assessment work (though could be incorporated in future assessments). The Octagon includes a domain for finance, an area not explicitly covered by the other approaches and omitted by the ESSENCE framework.

**Table 3.** The ESSENCE principles and comparison with Octagon Model.

ESSENCE principles	Octagon Model equivalent domains
Collaboration and communication	Implementation of activities
Understanding local context and existing research capacity	Working environment, Right skills in relation to activities
Local ownership and support	Working environment, Target groups
Ensure monitoring and evaluation	Broadly encompassed by Octagon overall
Robust research governance and effective leadership	Right skills in relation to activities, Structure and organization of activities, Basic values and identity, Systems for financing and administration
Strong support, supervision and mentorship structures	Right skills in relation to activities
Plan for long-term and ensure flexibility and continuity	Basic values and identity, Relevance

Note: Adapted from: ESSENCE on Health Research (2014).

These differences demonstrate the strengths and limitations of each framework; ESSENCE provides a set of broad principles of capacity development while the Octagon contains specific domains and a method for systematically evaluating the status of specific ethics activities and capabilities. Thus, we propose the Octagon model as a complementary tool for building research capacity.

## Discussion

In addition to the specific lessons regarding the evaluation process presented above, there are also several areas that need strengthening with institutional partners. Providing future partners timely feedback is key. Understanding the institutional leaders’ perceptions of the evaluation process is critical for shaping future partnerships; this will allow us to adapt the process in order to be most helpful for the partnering institutions. Whether this feedback should be incorporated into the baseline assessment or as part of a follow-up visit needs to be determined.

The three case studies also demonstrated the value of the external Octagon score, a component that should be retained in future assessments. In each of the three cases, the numerical scores across most domains were higher in the internal assessments (conducted by local institution faculty) than those given by the external (FABTP) assessment team. Though neither score is meant to be considered “correct”, these differences can be helpful catalysts for conversations between partners as they bring areas of need to the attention of the institution staff. Both scores should be included in a final evaluation. Understanding partner perceptions of both the internal and the external scores will help shape future assessments.

Moving forward, we hope to develop new methodological tools to help improve the evaluation processes. An implementation manual is under development to provide detailed tools and guidance for others seeking to assess institutional research ethics systems, particularly in LMICs. The manual will provide a framework and instructions to help guide assessors through the assessment process. Future efforts to conduct longitudinal case studies in this and related areas of capacity development are also needed.

## Conclusion

We hope that staff at other institutions can learn from the case studies presented previously and apply the Octagon framework to evaluate their own research ethics infrastructure. We present several suggestions that we hope will aid groups planning to conduct assessments of their own institutional research ethics capacity. As discussed, there are certainly limitations to the Octagon Model including the subjective nature of the score and an inability to compare across institutions. Despite these limitations, and though we continue to learn how to best implement the Octagon, it has provided a multi-dimensional framework for assessing institutional research ethics capacity. Understanding the lessons and limitations presented in this paper will help improve future assessments.

## Ethical approval

This work was reviewed and deemed exempt by the Institutional Review Board of the Johns Hopkins Bloomberg School of Public. The assessments were also approved by the review boards of each partnering institution.
